# Supplementary material for: Factors Influencing Pharmacokinetics of Tamoxifen in Breast Cancer Patients: A Systematic Review of Population Pharmacokinetic Models
Source: Biology (Basel). 2022 Dec 28;12(1):51. doi: 10.3390/biology12010051 (PMC9855885; doi:10.3390/biology12010051)
Supplement: Supplementary file 1 [file biology-12-00051-s001.zip › biology-2075092-supplementary.pdf]

Supplementary Table 1. Search Strategy

Database	Search Strategy	No. of Article
PUBMED	(Pharmacokinetics OR Population pharmacokinetics OR PopPK OR NONMEM) AND (Tamoxifen) AND (Breast neoplasms OR Breast Cancer)	724
Web of Science	(Pharmacokinetics OR Population pharmacokinetics OR PopPK OR NONMEM) AND (Tamoxifen) AND (Breast neoplasms OR Breast Cancer)	551
SCOPUS	(Pharmacokinetics OR “Population pharmacokinetics” OR PopPK OR NONMEM) AND (Tamoxifen) AND (“Breast neoplasms” OR “Breast Cancer”)	736
